# Behavioural Outcome in Children with Congenital Adrenal Hyperplasia: Experience of a Single Centre

**DOI:** 10.1155/2014/483718

**Published:** 2014-04-01

**Authors:** Arini Nuran Idris, Viji Chandran, Syed Zulkifli Syed Zakaria, Rahmah Rasat

**Affiliations:** ^1^Department of Paediatrics, Hospital Putrajaya, Pusat Pentadbiran Putrajaya, Presinct 7, 62250 Putrajaya, Malaysia; ^2^Department of Paediatrics, Universiti Kebangsaan Malaysia Medical Centre (UKMMC) Jalan Yaacob Latiff, Bandar Tun Razak, Cheras, 56000 Kuala Lumpur, Malaysia

## Abstract

The aim of this study was to determine the behavioral outcome in children with CAH and to identify the risk factors that may influence it. Participants (aged 6–18 years) included 29 girls and 20 boys with CAH and unaffected siblings (25 girls and 17 boys). Psychological adjustment was assessed with parent reports on the Child Behavior Checklist (CBCL). Information about disease characteristics was obtained from medical records. Our study reveals that there was higher incidence of parent-reported problem of anxious/depressed and withdrawn/depressed behaviours, somatic complaints, social, thought, and attention problems, and rule-breaking, aggressive, internalizing, and externalizing behaviour among children with CAH compared to controls. The prevalence of internalizing behaviour problems was higher in CAH boys compared with that of controls. Psychosocial adjustment of girls with CAH was found to be similar to unaffected female controls and was within the normal population range. Family income may be associated with behavioral outcome. Glucocorticoid dose may reflect disease severity which may be associated with behavioral outcome. We conclude that internalizing behavioral problem was prevalent among boys with CAH reflecting maladaptive adjustment in coping with chronic illness. This highlighted the importance of psychological and social support for the patients and their families.

## 1. Introduction

Congenital adrenal hyperplasia (CAH) consists of a family of defects in the synthesis of steroid hormones in the adrenal cortex. It is caused by a defect in the 21-hydroxylase gene (*CYP21*) in more than 90% of the cases [1, 2]. The enzyme deficiency results in impaired synthesis of cortisol and aldosterone. The low cortisol level results in increased production of ACTH by the pituitary, which causes increased synthesis of steroid precursors, resulting in high androgen levels. The androgen excess is present from early embryogenesis and results in varying degrees of virilization of the external genitalia in girls.

Girls with CAH are frequently studied in research focused on exploring the influence of atypical sex hormone exposure during brain development [3–5]. Prenatal androgen excess associated with masculinization of the genitalia is believed to influence the development of regions of the brain responsible for sex differences in behavior [6–9]. CAH has been utilized in research to study the influence of early androgen exposure on behaviors exhibiting sex-related variability, in particular psychosexual differentiation (i.e., gender identity, gender role, and sexual orientation) and sex differences in neurocognitive function [10–18].

Optimization of treatment aimed at achieving appropriate physical growth and maximizing good psychological adjustment is difficult. After birth, glucocorticoid and mineralocorticoid substitution decreases ACTH levels and thereby androgen production. This will avoid further masculinization of the female genitalia. Traditional practice of early surgery in girls was based on the assumption that this will lead to better psychosocial and psychosexual adjustment [2]. However, there is limited evidence available to support this practice. Recent reports have highlighted concern regarding possibility of early surgery causing emotional problems rather than preventing it [19, 20].

Lifelong medication and regular clinic visits are inevitable for CAH cases. The general consequences of chronic illness and specific consequences of androgen excess may interfere with normal psychosocial development.

Behavioral studies in children with congenital adrenal hyperplasia (CAH) may provide information that would help us determine whether any intervention can be instituted to minimize long-term adverse behavioral outcome. Currently, we do not have any objective information on behavioral outcome of children with CAH followed up in our centre. In order to address the question, we carried out this study to further investigate the behavioral outcome in children with CAH in UKMMC. For this purpose, we compared their behavior with that of their normal siblings who make up for the controls. We did not include assessment of psychosexual outcome in this study.

## 2. Research Objectives and Hypothesis

The general objective of this study was to determine the status of the behavioral outcome in children with CAH. Two specific objectives of this study were to compare the behavioral outcome in children with CAH to a normal control group and to identify features that may influence the behavioral outcome in children with CAH. The study was based on the hypotheses that there is no adverse behavioral outcome in children with CAH.

## 3. Subjects and Methods

### 3.1. Subjects

The study was carried out between April 2011 and January 30, 2012, and was approved by the Ethical Committee of the UKMMC. All CAH patients aged between 6 and 18 years (*N* = 65) who attended pediatric endocrine clinic, Universiti Kebangsaan Malaysia Medical Centre (UKMMC), were invited for inclusion into this study. Patients with mental retardation due to central nervous system infection, trauma, and other CNS conditions and patients with syndrome were excluded, *N* = 1. Children with CAH whose parents refused to participate in the study were not included in this study, *N* = 16. Finally, there were 49 CAH patients, males, *N* = 20, and females, *N* = 29, agreed to participate. The control group comprised of the 42 normal siblings of patients, males, *N* = 17, and females, *N* = 25. Since not all patients had a same age and sex control, relatives of male and female patients were combined to form male and female control groups, respectively. The majority of the CAH patients in our study were Malay ethnic, 80% (39/49); the remainder was comprised of Chinese 12% (6/49) and Indian 8% (4/49). The controls consisted of 74% Malay (31/42), 7% Chinese (3/42), and 19% Indians (8/42). Written informed consent was obtained from parents or legal guardians of all patients before participation. When informed consent was obtained, questionnaires were distributed to and self-administered by the parents. The completed questionnaires were returned before leaving the clinic.

Socioeconomic data were gathered during clinic interview. Background clinical data were collected from medical records including age at diagnosis, clinical and biochemical presentation at diagnosis, sex assignment, and type and age of genitoplasty. The levels of 17-hydroxyprogesterone (17-OHP) and testosterone, types and doses of glucocorticoids, and height standard deviation scores (SDs) closest in time to CBCL testing were recorded. Height SDs were calculated using the following formula, height SDs = (observed value − median value of the reference population)/standard deviation value of reference population. The median and SD values from US reference population were used in our study because these values were not yet established in our country [21]. Prader ratings were used to describe masculinization of the external genitalia at diagnosis.

Analysis of genetic mutations was not available for the patients. Based on clinical and biochemical characteristics at presentation, all patients in this study were grouped into salt wasting (SW) and simple virilising (SV) CAH. Medical treatment was started for SW patients at a median age of 0.5 months (range 0.1–2.0) and for the SV patients at 27 months (range 0.3–72). Patient characteristics were summarized in Table 1. All SW CAH had hyponatraemic episodes (less than 130 nmol/L) and elevated renin or at least one episode of hypotension or shock during early infancy. In the SV group, no hyponatremia was noted in early childhood. There were 3 boys with SV CAH who presented with precocious pseudopuberty including tall stature, phallic enlargement, and pubic hair development. There were 6 girls with SV CAH whose parents reported presence of clitoromegaly since birth but were lost to follow-up initially and later returned to medical attention because of progressive enlargement of the clitoris and tall stature. At diagnosis, all SV CAH patients had raised basal or unstimulated levels of 17-OHP and testosterone for age. All SW CAH patients had high basal levels of 17-OHP and raised testosterone and PRA levels for age. All girls with CAH had 46,XX chromosomal karyotype. Mean age of genitoplasty was 2.7 years (range 1–6.2). Genitoplasty was not done in 4 girls (3 SW and 1 SV) because parents opted to wait until the children were older before considering surgery.

The power of the study was back-calculated using the PS2 program. Using alpha value of 0.05, to find a difference in mean *T* scores of 10 with ratio control to patient being 0.85 and *n* of 49, the power of the study was obtained at 0.85 for the first specific objective of the study.

### 3.2. Questionnaire

The Child Behaviour Check List questionnaire (CBCL) was used in the study [22]. The questionnaire was a widely used instrument that assesses behavioral problems and social competence of children between the age 6 and 18 years. The CBCL form was widely used worldwide to evaluate children with chronic diseases. It had been translated to multiple languages and was validated for use.

In this study, the Bahasa Melayu (BM) version of the CBCL was used as most parents feel more comfortable to answer the questions in the national language. The BM version has been used in Malaysia for studies relating to child behaviour; however, the validation process results were yet to be published [22].

The BM version had been translated and back translated with permission, by a team of psychiatrists and doctors. Two front translations and back translations were done including translations by the professional translators. An internal validation study was carried out in 2008 by a team of psychiatrists and psychologists involving parents of children aged 6 to 18 years (*n* = 30) attending pediatric clinic follow-up in UKMMC. A language fluency test was carried out prior to the test and only bilingual parents were included. Subjects were asked to complete both the English and BM versions. The internal reliability was between 0.7–0.9 (Cronbach alpha) [22, 23]. The final version produced was used in this study. Permission was obtained from the organization which produced the questionnaire (ASEBA), the authors who translated the BM version, and the authors who carried out the pretest study.

The CBCL consisted of 118 items or questions. The parents answered the questions based on their child's behavior for the past 6 months. Parents rated their child and chose either 0 for none, 1 for sometimes, and 2 for always. From various combinations of the items or questions, the behavioral problem scale was derived. The scale consisted of 3 summary scales and 8 syndrome scales. The 8 syndrome scales included “withdrawn/depressed problem,” “somatic complaint problem,” “anxious/depressed problem,” “social problem,” “thought problem,” “attention problem,” “rule-breaking problem,” and “aggressive problem.” The 3 summary scales were formed from summation of scores of the certain syndrome: internalizing behavioral problem (consisted of score combination of withdrawal, somatic complaints, withdrawn/depressed, and anxiety/depressed problems), externalizing behavioral problem (formed by score combination of aggressive and rule-breaking behavior problems), and total problems (score combination of all items). Internalizing summary scale comprised problems that were mainly within the self and externalizing summary scale comprised problems that mainly involved conflicts with other people and with their expectations for the child.

The information from the questionnaires was entered into a CBCL software program which in turn calculated the raw scores, *T* scores, and the score percentile. *T* scores of the CBCL summary and syndrome scales were used for analysis. *T* scores for summary scales which were above 63 (>97th percentile of the normative sample) and for syndrome scales above 69 (>97th percentile of the normative sample) were considered to be clinical range scores. The clinical range scores indicated that the person who completed the CBCL reported enough problems to be of clinical concern. These norms were based from another country where CBCL was originally developed. The clinical range scores based on Malaysian national sample of normal children were not available at the time this study.

### 3.3. Statistical Analysis

Data was analyzed using the IBM SPSS version 21. Differences in demographic data between groups were tested using independent *t*-tests for comparison of means for continuous variable and *χ* ^2^ test for categorical variables. A two-way MANOVA was used to analyze the interaction between multiple independent and dependent variables. For identification of risk factors, bivariate analysis (Pearson's correlation) was performed and those factors found to be significant were included in the simple linear regression model. Statistical significance was assumed when *P* < 0.05. In view of the importance of finding of abnormal results and the small sample size, no correction was done for multiple testing.

## 4. Results

### 4.1. Patient Cohort and Control

Case and control groups were similar with regard to mean age at study entry, mean family income per month, ethnicity, and gender distribution as shown in Table 2.

Similarly, there were no significant differences between the male and female CAH compared to male and female controls, respectively, with regard to the demographic data. The mean age of male CAH (*N* = 20) was 11.6 years (SD = 3.2) versus mean age of male control (*N* = 17) being 12.8 years (SD = 4.3) (*t* statistic, 0.9; mean difference, 141; 95% CI, −1.4, 3.8).

Mean family income per month of male CAH was RM 4300 (SD = 3640) versus control RM 4441 (SD = 3091) (*t* statistic, 0.1; mean difference, 1.4; 96% CI, −2135, 2418). Ethnic distribution of the male CAH (*N* = 20) was Malay, *N* = 14 (70%), other ethnic groups, *N* = 6, (30%) versus controls (*N* = 17) Malay, *N* = 13 (76%), and others, *N* = 4 (24%) (*P* = 0.7).

Parental marital status of male CAH (*N* = 20) was married, *N* = 18 (90%), single, *N* = 2, (10%) versus controls (*N* = 17), married, *N* = 16 (94%), and single, *N* = 1 (17%) (*P* = 1.0).

Mean age of female CAH (*N* = 29) was 11.9 years (SD = 3.4) versus female controls (*N* = 25) being 12.2 years (3.8) (*t* statistic, 0.35; mean difference, 0.3; 95% CI, −1.7, 2.4).

Mean family income per month of female CAH (*N* = 29) is RM 4441 (SD = 3087) versus controls (*N* = 25) being RM 4112 (2883) (*t* statistic, −0.4; mean difference, −329; 95% CI, −196, 1302). Ethnic distribution of the female CAH (*N* = 29) was Malay, *N* = 24 (83%), others, *N* = 5 (17%) versus controls (*N* = 25), *N* = Malay, 18 (72%), and others 7 (28%) (*χ* ^2^ statistic, 0.9; *P* = 0.3). Parental marital status of female CAH (*N* = 29) is married, *N* = 27 (93%), single, 2 (7%) versus controls (*N* = 25) married, *N* = 25 (100%), and single, 0 (0%) (*P* = 0.5).

### 4.2. Comparison of Mean *T* Scores for Summary Scales between CAH and Control Groups

To test the hypothesis that disease (CAH), gender, and the interaction between CAH and gender had a significant impact on internalizing and externalizing mean *T* scores, a two-way MANOVA was conducted. A significant multivariate main effect for CAH was found, Wilk's Lambda = 0.769, *F* (2, 86) = 11.011, *P* < 0.001, partial eta squared = 0.204. Power to detect the effect was 0.990. There was no significant main effect for gender, Wilk's Lambda = 0.961, *F* (2, 86) = 1.741, *P* = 0.181, partial eta squared = 0.039. Power to detect the effect was 0.356. There was no significant interaction effect of CAH and gender, Wilk's Lambda 0.982, *F* (2, 86) = 0.776, *P* = 0.463, partial eta squared = 0.018, and power to detect the effect was 0.178. Given the significance of the multivariate main effect of the CAH, the univariate main effects were examined. Significant main effects for CAH were obtained for *T* scores for internalizing scale, *F* (1, 87) = 17.511, *P* < 0.001, partial eta squared = 0.168, power = 0.985, and externalizing scale, *F* (1, 87) = 20.28, *P* < 0.001, partial eta squared 0.189, power = 0.994. Significant mean differences in *T* scores were obtained between the CAH cases and controls for internalizing scale (mean difference = 9.05; 95% CI = 4.751, 13.346; *P* < 0.001) and externalizing scale (mean difference 8.478; 95% CI = 4.736, 12.219; *P* < 0.001) as shown in Table 3. All mean *T* scores fell within normal range of population norms.

### 4.3. Prevalence of CAH Patients with Clinical Range Scores

A chi-square test of independence was performed to examine the proportion of CAH cases with clinical range *T* scores for internalizing and externalizing scales compared to that of controls (separated by gender). The analysis revealed that significantly higher proportion of CAH males compared to controls had clinical range scores for internalizing scale, *χ* ^2^ (1, *N* = 20) =6.13, *P* = 0.017. The CAH males did not differ significantly from control in the externalizing scale. The prevalence of patients with clinical range scores for internalizing and externalizing scales in CAH females did not differ significantly from that of controls. These results were shown in Table 4.

### 4.4. Comparison of Mean *T* Scores for Syndrome Scales between CAH and Control Groups

The two-way MANOVA was conducted to test the hypothesis that CAH, gender, and the interaction between CAH and gender had significant influence on mean *T* scores of 8 syndrome scales. A significant main effect was found for CAH, *F* (8, 80) = 3.118, *P* = 0.004, partial eta squared = 0.238, power = 0.949, but not for gender, *F* (8, 80) = 1.603, *P* = 0.137, power = 0.670, and not for their interactions *F* (8, 80) = 0.830, *P* = 0.579, power = 0.359.

Given the significance of the multivariate main effect of CAH, the univariate main effects were examined. Significant main effects for CAH were obtained for mean *T* scores for anxious/depressed syndrome, *F* (1, 87) = 14.303, *P* < 0.001, withdrawn/depressed syndrome, *F* (1, 87) = 11.459, *P* < 0.001, attention problems scale, *F* (1, 87) = 16.474, *P* < 0.001, thought problems scale, *F* (1, 87) = 15.364, *P* < 0.001, social problems scale *F* (1, 87) = 18.280, *P* ≤ 0.001, aggressive behavior scale *F* (1, 87) = 14.728, *P* < 0.001, and total problems scale, *F* (1, 87) = 28.065, *P* < 0.001. There were also significant main effects for CAH for somatic complaints and rule-breaking behavior scales.  Table 5 showed significant mean differences (*P* = 0.001) in *T* scores obtained between the CAH cases and controls for anxious/depressed, withdrawn/depressed, attention problems, thought problems, social problems, and aggressive behavior scales. The mean differences were also significant in the somatic complaints and rule-breaking behaviour scales. None of the mean *T* scores were clinical range scores.

### 4.5. Proportion of CAH Patients with Clinical Range Scores for Syndrome Scales Compared to That of Controls

A chi-square test of independence was performed to examine the proportion of CAH cases with clinical range scores for syndrome scales compared to that of controls (separated by gender).  Table 6 illustrated that there was no significant difference in the proportion of male or female patients with clinical range scores for all 8 syndrome scales compared to that of controls, respectively.

### 4.6. Associations

To evaluate associations between psychosocial adjustment and other factors such as age at study entry, family income, and disease characteristics, Pearson's correlation and simple linear regression analysis were performed. The results were illustrated in Tables 7, 8, 9, and 10. There was a significant (linear) negative correlation between family income and mean *T* scores for internalizing, *r* = −0.30, *P* = 0.003, and externalizing, *r* = −0.30, *P* = 0.005, as highlighted in Table 7. This indicated that lower income was associated with higher the mean *T* scores (poorer psychosocial adjustment).

Table 10 highlighted that family income significantly predicted mean *T* scores for internalizing, *b* = 1.05, *t* = 3.12, *P* = 0.003, and externalizing scales, *b* = 1.19, *t* = 3.22, *P* = 0.002. Family income also explained a proportion of variance in mean *T* scores for internalizing, *R* ^2^ = 0.18, *P* = 0.003, and externalizing scale, *R* ^2^ = 0.18, *P* = 0.002.

Glucocorticoid dose was positively correlated with mean *T* scores for internalizing, *r* = 0.43, *P* = 0.002, and externalizing scales, *r* = 0.41, *P* = 0.003, as shown in Table 8. This suggested that higher glucocorticoid dose was associated with higher mean *T* scores for the 2 summary scales (poorer psychosocial adjustment).

Table 10 illustrated that glucocorticoid dose significantly predicted mean *T* scores for internalizing, *b* = 1.19, *t* = 3.22, *P* = 0.002, and externalizing scales, *b* = 0.95, *t* = 3.09, *P* = 0.003. A proportion of variance in mean *T* scores for internalizing scale was explained by glucocorticoid dose, *R* ^2^ = 0.18, *P* = 0.002, and externalizing scale, *R* ^2^ = 0.17, *P* = 0.003.

Table 9 showed that there were no significant correlations found between serum testosterone of CAH girls at study entry, Prader rating and age at clitoroplasty or vaginoplasty with mean *T* scores of either internalizing or externalizing summary scales.

## 5. Discussion

Our overall results revealed that the parents of the CAH children reported a higher incidence of anxious/depressed behavior, withdrawn/depressed behavior, somatic complaints, social problems, thought problems, attention problems, rule-breaking behavior, and aggressive behavior among their children than did the mothers of the control patients. However, mean *T* scores of the children with CAH fell within the normal ranges of the normative sample on each of these summary and syndrome scales. The prevalence of internalizing behaviour problems, included combination of anxious/depressed and withdrawn/depressed behaviors and somatic complaints were higher in CAH boys compared with those of controls. Psychosocial adjustment of girls with CAH was found to be similar to unaffected females controls and was within the normal population range. Our study showed that family income and glucocorticoid dose may be associated with higher parent-reported problem scores for internalizing and externalizing behaviour.

Studies on behavioural outcome in children with CAH were scarce. Majority of previous studies concentrated on gender issues and psychosexual adjustment mainly involving females with CAH. Our results of boys with CAH having more behavioral difficulties compared to controls especially in the internalizing scale contradicted findings of other studies of good overall psychosocial adjustment in boys [24–26]. It is not surprising that boys with CAH were reported to have a greater number of anxious/depressed, withdrawn/depressed behaviors, and somatic complaints, since they have chronic illness, seen in endocrine clinic 3-4 times each year, receive daily medication, and are closely monitored by their parents and physician if they become ill.

Similarities between girls with CAH and controls in psychosocial adjustment were consistent with other studies finding good overall adjustment in females with CAH [24–28]. Previous studies have suggested that CAH females have higher energy and aggression levels, participation in sports, and interest in masculine physical games and behaviors [14, 15, 17, 29]. However, our findings showed that the responses of the girls with CAH were no different than those of the community and sibling controls. Aggression was assessed on a summary scale of the CBCL. On this scale, mothers were asked to rate their child in terms of such aggressive behaviors as fighting, demanding attention, swearing, and threatening people. The girls with CAH and the controls fell within the normal ranges on this subscale. Money and Schwartz found that CAH females do not establish close psychological relationships with females peers [30]. In light of this finding, we expected that the girls with CAH in this study would have high scores on CBCL social problems scale. However, the mean responses of the CAH and the control group fell within the normal ranges on this measure.

The finding of psychologically well-adjusted girls with CAH may be attributed to successful genital surgery, closely monitoring urinary plasma steroid, and appropriate emotional support [24]. Other studies suggested that chronic illness and other difficult life experiences have only temporary effects on adjustment. For example, patients suffering from cancer and sickle cell disease and boys with hypospadias who had undergone surgery were shown to have psychological adjustment that was no different from the normal children [31–33].

Our study showed that socioeconomic status may contribute to behavioral problem in CAH children. Zebrack et al. studied the 5736 adult survivors of acute leukemia, Hodgkin's disease, and non-Hodgkin lymphoma and 2565 sibling controls. They reported that socioeconomic factors may be associated with more depressive and somatic distress in the adult cancer survivors [34]. Leukaemia/lymphoma survivors had a significantly higher symptomatic depression and somatic complaints. However, the authors were not able to conclude whether there were any direct relationship between psychological distress and socioeconomic status of the family.

From the National Health and Morbidity Survey (NHMS III 2006), children and adolescent from households with incomes lower than RM 3000 have significantly higher psychiatric morbidity [35]. We are uncertain whether this finding could be extrapolated to our study findings.

High glucocorticoid dose at study entry appeared to be associated with higher parent-reported problems for externalizing and internalizing behavior. High dose of glucocorticoid (>20 mg/m^2^/day) had been reported to be associated with clinical disease severity [36]. Therefore, the observed correlation between glucocorticoid with behavioral problems may actually indicate the relationship between disease severity and poor behavioral outcome.

Although behavioural problems did not appear to depend on disease characteristics (such as mean age at diagnosis, serum 17-OHP at study entry, height SDS, serum testosterone at study entry, Prader rating, and age of genitoplasty), sample size was small to make conclusion about psychosocial outcome and disease associations.

The finding of increased prevalence of internalizing behaviour problem in CAH boys reflected responses to coping with CAH in general. It was an essential finding because it highlighted the importance of the overall care of patients with CAH. Psychological screening could be useful in identifying CAH patients at high risk for psychosocial maladjustment so that further psychological evaluation and intervention could be provided early to minimize the problem. Patients who are coming from lower socioeconomic class may benefit from early referral to and regular review by the medical social services.

Future multicenter study involving CAH children in Malaysia is encouraged to ensure larger sample size to produce a rigorous study methodology. In addition, further studies should concentrate on masculinizing behavior of Malaysian girls with CAH or psychosexual outcome of Malaysian CAH females. These studies should include Malaysian children with other chronic illness such as obesity or diabetes mellitus as controls because they share very similar life experiences that accompany their life respective chronic illnesses.

Our study has some methodological limitations. One of the limitation was as to whether the patients studied likely represent the population with CAH given that 16 (12 boys and 4 girls) out of 65 eligible patients refused to participate in the study. All 4 girls had genital surgery at an early age. History of school refusal was reported in 2 girls, 1 girl was a school drop-out, and 1 girl had gender identity problem. Furthermore, retrospective collection of medical and surgical information of varied quality resulted in inability to evaluate outcome in relation to quality of genital surgery, number of genital surgery, and number of genital examinations. Another limitation was that the information on the children's behavior was provided by their parents or legal guardians, and completing information from the children themselves and/or data from behavior observation would be desirable. Normal range of *T* scores from US population norms were used in this study and, ideally, normal *T* scores for Malaysian population norms should be used as reference if available.

Our findings of adverse psychological adjustment in patients with CAH boys reflected consequences of chronic disease or maladjusted coping behavior to chronic disease in general. Psychological adjustment was not compromised in girls with CAH. Conclusion that the psychological adjustment was independent of other studied factors (such as mean age at diagnosis, serum 17-OHP at study entry, height SDS, serum testosterone at study entry, Prader rating, and age of genitoplasty) should be made with caution because of the limitations of this study. Family income may be associated with worse behavioural outcome. Glucocorticoid dose may be influenced by disease severity which in turn may affect the behavioral outcome. Our findings underscored the importance of psychological and social support for the patients and their families. We recommend that children with CAH should undergo psychological screening and those found to have abnormal screening results may benefit from further psychological evaluation and intervention.

## Figures and Tables

**Table 1:** Disease characteristics of CAH cases.

	Male *N* = 20	Female *N* = 29
Age (in months) at starting treatment^a^	1.0 (1.5)	0.5 (0.6)
Age in years at study entry	11.6 (4.3)^a^	11.9 (3.4)^a^
	11.5 (6–18)^b^	12.0 (6–17)^b^
Presentation at diagnosis		
Salt wasting features^c^	17 (85)	23 (79)
No salt wasting features^c^	3 (15)	6 (21)
Ambiguous genitalia^c^	0	29 (100)
Hypertension^c^	0	0
Height SDS at the time of CBCL^a^	−1.1 (1.6)	−1.4 (2.2)
Serum 17-OHP in nmol/L at the time of CBCL^a^	46.0 (182.1)	8 (131.1)
Glucocorticoid dose in mg/m^2^/day near the time of CBCL^a^	17.3 (4.9) (CI: 15–19.6)	14.4 (2.4) (CI: 13.9–15.7)
Prader rating^c^		
1		4 (14)
2		8 (28)
3		14 (48)
4		3 (10)
Genitoplasty^c^		
Nil		4 (14)
Clitoroplasty		11 (38)
Clitoroplasty and vaginoplasty		14 (48)
Age in years at the first genitoplasty^a^		2.3 (2.7)
Serum testosterone in nmol/L of CAH girls at the time of CBCL^a^		0.7 (3.3)

^a^Mean (standard deviation).

^b^Median (range).

^c^Frequency (%).

CI: confidence interval.

**Table 2:** Sociodemographic characteristics of study sample.

Variable	*N*	Case	Control	Mean difference (95% CI)	*t* statistic^a^ (df)	*χ* ^2^ statistic^b^ (df)	*P* value^d^
Mean age at study entry in years^a^ (SD)							
Case	49	11.8 (3.8)	—	0.7 (0.8–2.2)	0.871 (89)	—	0.386
Control	42	—	12.5 (3.6)				
Gender^b^ frequency (%)							
Male	37	20 (54)	17 (46)	—	—	0.00 (1)	0.974
Female	54	29 (54)	25 (46)				
Ethnicity^b^ frequency (%)							
Malay	69	38 (55)	31 (45)	—	—	0.71 (1)	0.678
Others	22	11 (50)	11 (50)				
Mean family income in RM^a^ (SD)							
Case	49	4383.7 (3287.6)	—	−138.4 (−1444.6, 1169.7)	−210 (89)		0.834
Control	42	—	4245.2 (2936.5)				
Parental marital status^c^ Frequency (%)							
Married	86	45 (52)	41 (48)		—	—	0.369
Single	5	4 (80)	1 (20)				

^a^Independent *t*-test.

^b^ *χ* ^2^ test for independence.

^c^Fisher's exact test.

^d^Significant *P* value <0.05.

SD: standard deviation; CI: confidence interval; RM: Ringgit Malaysia.

**Table 3:** Mean *T* scores and mean differences for summary scales for cases and controls.

	Case *N* = 49	Control *N* = 42	Mean difference (95% CI)	*P* value^a^
Internalizing				
Mean	59.0	50.0	9.0	<0.001*
SD	(10.5)	(9.7)	(4.8, 13.3)	
Externalizing				
Mean	57.1	48.7	8.5	<0.001*
SD	(8.7)	(9.1)	(4.7, 12.2)	

^a^ANOVA.

SD: standard deviation; CI: confidence interval.

*Significant *P* value <0.05.

Clinical range *T* scores >63.

**Table 4:** Proportions of CAH patients with clinical range scores on internalizing and externalizing scales compared to the controls.

	Male				Female			
	Case *N* = 20	Control *N* = 17	*χ* ^2^	*P* value^a^	Case *N* = 29	Control *N* = 25	*χ* ^2^	*P* value^a^
Internalizing frequency (%)	10 (50)	2 (12)	6.13	0.017	5 (17)	4% (1)	2.38	0.200
Externalizing frequency (%)	6 (30)	1 (6)	3.84	0.097	3 (10)	4% (1)	0.78	0.620

^a^Fisher's exact test.

Significant *P* value <0.05.

**Table 5:** Mean *T* scores and mean differences for syndrome scales for cases and controls.

	Case *N* = 49 Mean *T* score (SD)	Control *N* = 42 Mean *T* score (SD)	Mean difference (95% CI)	*P* value^a^
Anxious-depressed	57.8 (7.9)	52.8 (3.7)	5.0 (1.1, 8.9)	<0.001
Withdrawn-depressed	59.2 (8.5)	54.1 (5.0)	5.1 (0.7, 9.6)	0.001
Somatic	61.4 (10.4)	56.0 (7.3)	5.4 (−0.3, 11.1)	0.006
Social	59.7 (6.8)	54.4 (4.2)	5.2 (1.6, 8.8)	<0.001
Thought	58.0 (8.0)	52.8 (4.0)	5.2 (1.3, 9.1)	<0.001
Attention	60.9 (9.2)	54.2 (5.2)	6.7 (1.8, 11.5)	<0.001
Rule-breaking	57.2 (7.5)	53.5 (4.9)	3.7 (−0.4, 7.8)	0.009
Aggressive	58.4 (7.8)	53.2 (4.7)	5.2 (1.2, 9.2)	<0.001

^a^ANOVA.

SD: standard deviation; CI: confidence interval.

Statistically significant *P* value <0.05.

Clinical range *T* scores >69.

**Table 6:** Percentage of CAH patients with clinical range scores on syndrome scales compared to the controls.

	Male				Female			
	Case *N* = 20	Control *N* = 17	*χ* ^2^	*P* value^a^	Case *N* = 29	Control *N* = 25	*χ* ^2^	*P* value^a^
Anxious/depressed % (*N*)	15% (3)	0% (0)	2.78	0.23	10% (3)	0% (0)	2.74	0.24
Withdrawn/depressed % (*N*)	20% (4)	6% (1)	1.57	0.35	14% (4)	0% (0)	3.72	0.11
Somatic % (*N*)	35% (7)	12% (2)	2.70	0.13	7% (2)	0% (0)	1.79	0.49
Social % (*N*)	5% (1)	0% (0)	0.87	1.00	7% (2)	0% (0)	1.79	0.49
Thought % (*N*)	10% (2)	0% (0)	1.80	0.49	0% (0)	0% (0)	—	0.11
Attention % (*N*)	15% (3)	0% (0)	2.78	0.23	14% (4)	0% (0)	3.73	0.34
Rule-breaking % (*N*)	0% (2)	6% (1)	0.21	1.00	3.5% (1)	0% (0)	0.89	1.00
Aggressive % (*N*)	25% (5)	0% (0)	4.91	0.05	3.5% (1)	0% (0)	0.87	1.00

^a^Fisher's exact test.

Significant *P* < 0.05.

**Table 7:** Correlations between psychological adjustment and age at study entry and family income in study subjects.

	*N*	Internalizing *r*, *P* value	Externalizing *r*, *P* value	Total *r*, *P* value
Age at study entry	91	0.07, ns	−0.14, ns	−0.07, ns
Family income	91	−0.30, 0.003*	−0.30, 0.005*	−0.38, <0.001*

*r*: Pearson's correlation coefficient; ns: not significant.

*Significant *P* value <0.05 (2-tailed).

**Table 8:** Correlations between psychological adjustment and disease characteristics in CAH patients.

	*N*	Internalizing *r*, *P* value	Externalizing *r*, *P* value	Total *r*, *P* value
Age at diagnosis	49	−0.03, ns	−0.02, ns	0.02, ns
Height SDs at study entry	49	0.00, ns	0.17, ns	0.06, ns
Dose of glucocorticoid at study entry	49	0.43, 0.002*	0.41, 0.003*	0.42, 0.003*
Serum 17-OHP at study entry	49	0.10, ns	0.02, ns	0.10, ns

*r*: Pearson's correlation coefficient; ns: not significant.

*Significant *P* value <0.05 (2-tailed).

**Table 9:** Correlations between summary scales *T* scores and disease characteristics in CAH patients.

	*N*	Internalizing *r*, *P* value	Externalizing *r*, *P* value	Total *r*, *P* value
Serum testosterone of CAH girls at study entry	29	−0.08, ns	0.13, ns	0.05, ns
Prader rating	29	−0.14, ns	0.02, ns	−0.01, ns
Age at clitoroplasty or vaginoplasty	25	−0.00, ns	−0.17, ns	−0.08, ns

*r*: Pearson's correlation coefficient; ns: not significant.

**Table 10:** Simple linear regression analysis on factors correlated with mean *T* scores for summary scales.

Independent variable	Dependent variable	*b* (95% CI *β*)	*t* statistic	*P* value	*F*	*r* ^2^
Family income *N* = 91	Internalizing	1.05 (0.39, 1.71)	3.12	0.003*	9.01	0.178
	Externalizing	1.19 (0.45, 1.93)	3.22	0.002*	8.48	0.181
Glucocorticoid dose *N* = 49	Internalizing	1.19 (0.45, 1.93)	3.22	0.002*	10.39	0.18
	Externalizing	0.95 (0.33, 1.57)	3.09	0.003*	9.55	0.17

*Significant *P* value <0.05.

CI: confidence interval.

## References

[1] White PC, Speiser PW (2000). Congenital adrenal hyperplasia due to 21-hydroxylase deficiency. *Endocrine Reviews*.

[2] New MI (2004). An update of congenital adrenal hyperplasia. *Annals of the New York Academy of Sciences*.

[3] Hines M, Brook C, Conway GS (2004). Androgen and psychosexual development: core gender identity, sexual orientation, and recalled childhood gender role behavior in women and men with congenital adrenal hyperplasia (CAH). *Journal of Sex Research*.

[4] Berenbaum SA, Duck SC, Bryk K (2000). Behavioral effects of prenatal versus postnatal androgen excess in children with 21-hydroxylase-deficient congenital adrenal hyperplasia. *Journal of Clinical Endocrinology and Metabolism*.

[5] Collaer ML, Hines M (1995). Human behavioral sex differences: a role for gonadal hormones during early development?. *Psychological Bulletin*.

[6] Beatty WW (1979). Gonadal hormones and sex differences in nonreproductive behaviors in rodents: organizational and activational influences. *Hormones and Behavior*.

[7] Ehrhardt AA, Epstein R, Money J (1968). Fetal androgens and female gender identity in the early-treated adrenogenital syndrome. *Bulletin of the Johns Hopkins Hospital*.

[8] Slijper FME (1984). Androgens and gender role behavior in girls with congenital adrenal hyperplasia (CAH). *Progress in Brain Research*.

[9] Dittmann RW, Kappes MH, Kappes ME (1990). Congenital adrenal hyperplasia I: gender-related behavior and attitudes in female patients and sisters. *Psychoneuroendocrinology*.

[10] Berenbaum SA, Holmes CS (1990). Congenital adrenal hyperplasia: intellectual and psychosexual functioning. *Psychoneuroendocrinology: Brain, Behavior, and Hormonal Interactions*.

[11] Resnick SM, Berenbaum SA, Gottesman II, Bouchard TJ (1986). Early hormonal influences on cognitive functioning in congenital adrenal hyperplasia. *Developmental Psychology*.

[12] Hampson E, Rovet JF, Altmann D (1998). Spatial reasoning in children with congenital adrenal hyperplasia due to 21-hydroxylase deficiency. *Developmental Neuropsychology*.

[13] Helleday J, Edman G, Ritzen EM, Siwers B (1993). Personality characteristics and platelet MAO activity in women with congenital adrenal hypreplasia (CAH). *Psychoneuroendocrinology*.

[14] Berenbaum SA, Hines M (1992). Early androgens are related to childhood sex-typed toy preferences. *Psychological Science*.

[15] Berenbaum SA, Snyder E (1995). Early hormonal influences on childhood sextyped activity and playmate preferences: implications for the development of sexual orientation. *Developmental Psychology*.

[16] Quadagno DM, Briscoe R, Quadagno JS (1977). Effect of perinatal gonadal hormones on selected nonsexual behavior patterns: a critical assessment of the nonhuman and human literature. *Psychological Bulletin*.

[17] Berenbaum SA (1999). Effects of early androgens on sex-typed activities and interests in adolescents with congenital adrenal hyperplasia. *Hormones and Behavior*.

[18] Zucker KJ, Bradley SJ, Oliver G, Blake J, Fleming S, Hood J (1996). Psychosexual development of women with congenital adrenal hyperplasia. *Hormones and Behavior*.

[19] Intersex Society of North American http://www.isna.org.

[20] Diamond M, Sigmundson HK (1997). Sex reassignment at birth: long-term review and clinical implications. *Archives of Pediatrics and Adolescent Medicine*.

[21] 2000 CDC Growth charts for United States: methods and development.

[22] Achenbach TM, Rescorla LA (2001). *Manual for ASEBA School-Age Forms & Profiles, Research Center for Children, Youth, & Families*.

[23] Abd Rahman FN, Mohd Daud TI, Nik Jaafar NR, Shah SA, Tan SM, Wan Ismail WS (2013). Behavioral and emotional problems in a Kuala Lumpur children's home. *Pediatrics International*.

[24] Gordon AH, Lee PA, Dulcan MK, Finegold DN (1986). Behavioral problems, social competency, and self perception among girls with congenital adrenal hyperplasia. *Child Psychiatry and Human Development*.

[25] Berenbaum SA, Bryk KK, Duck SC, Resnick SM (2004). Psychological adjustment in children and adults with congenital adrenal hyperplasia. *Journal of Pediatrics*.

[26] Berenbaum SA, Bryk KK, Duck SC (2010). Normal intelligence in female and male patients with congenital adrenal hyperplasia. *International Journal of Pediatric Endocrinology*.

[27] Hurtig AL, Rosenthal IM (1987). Psychological findings in early treated cases of female pseudohermaphroiditism caused by virilizing congenital adrenal hyperplasia. *Archives of Sexual Behavior*.

[28] Kuhnle U, Bullinger M, Schwarz HP (1995). The quality of life in adult female patients with congenital adrenal hyperplasia: a comprehensive study of the impact of genital malformations and chronic disease on female patients life. *European Journal of Pediatrics*.

[29] Berenbaum SA, Resnick SM (1997). Early androgen effects on aggression in children and adults with congenital adrenal hyperplasia. *Psychoneuroendocrinology*.

[30] Ehrhardt AA, Money J (1967). Progestin-induced hermaphroditism: I. Q. and psychosexual identity in a study often girls. *Journal of Sex Research*.

[31] Noll RB, Vannatta K, Koontz K, Kalinyak K, Bukowski WM, Davies WH (1996). Peer relationships and emotional well-being of youngsters with sickle cell disease. *Child Development*.

[32] Noll RB, Gartstein MA, Vannatta K, Correll J, Bukowski WM, Hobart Davies W (1999). Social, emotional, and behavioral functioning of children with cancer. *Pediatrics*.

[33] Mureau MAM, Slijper FME, Slob AK, Verhulst FC (1997). Psychosocial functioning of children, adolescents, and adults following hypospadias surgery: a comparative study. *Journal of Pediatric Psychology*.

[34] Zebrack BJ, Zeltzer LK, Whitton J (2002). Psychological outcomes in long-term survivors of childhood leukemia, Hodgkin’s disease, and non-Hodgkin’s lymphoma: a report from the childhood cancer survivor study. *Pediatrics*.

[35] The Third National Health and Morbidity Survey (NHMS III) http://www.nih.gov.my.

[36] Hall CM, Jones JA, Meyer-Bahlburg HFL (2004). Behavioral and physical masculinisation are related to genotype in girls with congenital adrenal hyperplasia. *Journal of Clinical Endocrinology and Metabolism*.

